# Epigenetic Regulation of Driver Genes in Testicular Tumorigenesis

**DOI:** 10.3390/ijms24044148

**Published:** 2023-02-19

**Authors:** Finn E. von Eyben, Karsten Kristiansen, Daniel S. Kapp, Rong Hu, Ovidiu Preda, Francisco F. Nogales

**Affiliations:** 1 Center for Tobacco Control Research, Birkevej 17, 5230 Odense, Denmark; 2 Laboratory of Genomics and Molecular Biomedicine, August Krogh Building Department of Biology, University of Copenhagen, Universitetsparken 13, 2100 Copenhagen, Denmark; 3 BGI-Research, BGI-Shenzhen, Shenzhen 518120, China; 4 Institute of Metagenomics, Qingdao-Europe Advanced Institute for Life Sciences, BGI-Qingdao, Qingdao 166555, China; 5 Department of Radiation Oncology, Stanford University, Stanford, CA 94305, USA; 6 Department of Pathology, Laboratory Medicine, University of Wisconsin Hospital and Clinics, Madison, WI 53792, USA; 7 Department of Pathology, San Cecilio University Hospital, 18071 Granada, CP, Spain; 8 Department of Pathology, School of Medicine, University Granada, 18071 Granada, CP, Spain

**Keywords:** differentiation, epigenetics, LIN28, NANOG, POU5F1, SOX2, testis cancer

## Abstract

In testicular germ cell tumor type II (TGCT), a seminoma subtype expresses an induced pluripotent stem cell (iPSC) panel with four upregulated genes, OCT4/*POU5F1, SOX17, KLF4,* and *MYC,* and embryonal carcinoma (EC) has four upregulated genes, OCT4/*POU5F1, SOX2, LIN28,* and *NANOG.* The EC panel can reprogram cells into iPSC, and both iPSC and EC can differentiate into teratoma. This review summarizes the literature on epigenetic regulation of the genes. Epigenetic mechanisms, such as methylations of cytosines on the DNA string and methylations and acetylations of histone 3 lysines, regulate expression of these driver genes between the TGCT subtypes. In TGCT, the driver genes contribute to well-known clinical characteristics and the driver genes are also important for aggressive subtypes of many other malignancies. In conclusion, epigenetic regulation of the driver genes are important for TGCT and for oncology in general.

## 1. Introduction: Epigenetics, Differentiation, and Testicular Germ Cell Tumors

**Figure 1:**
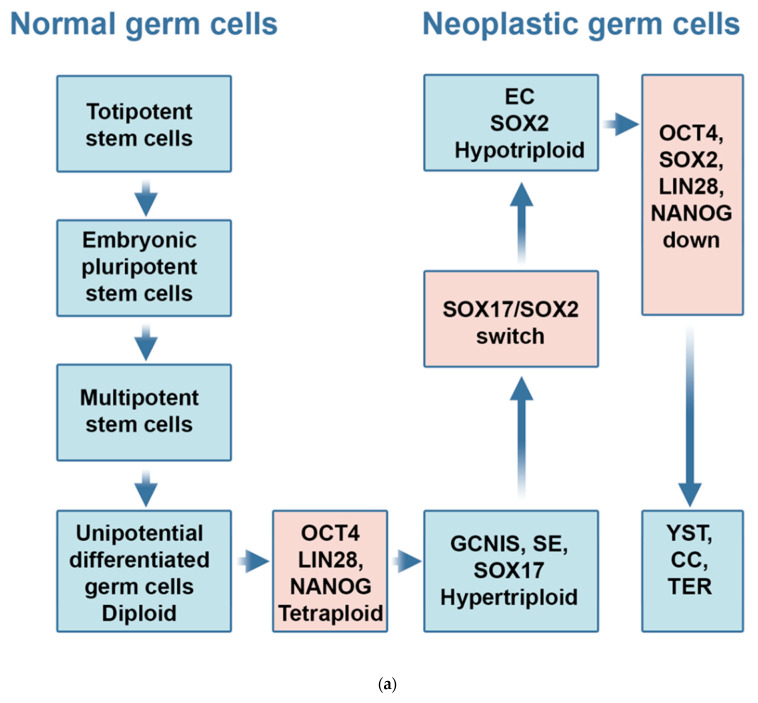

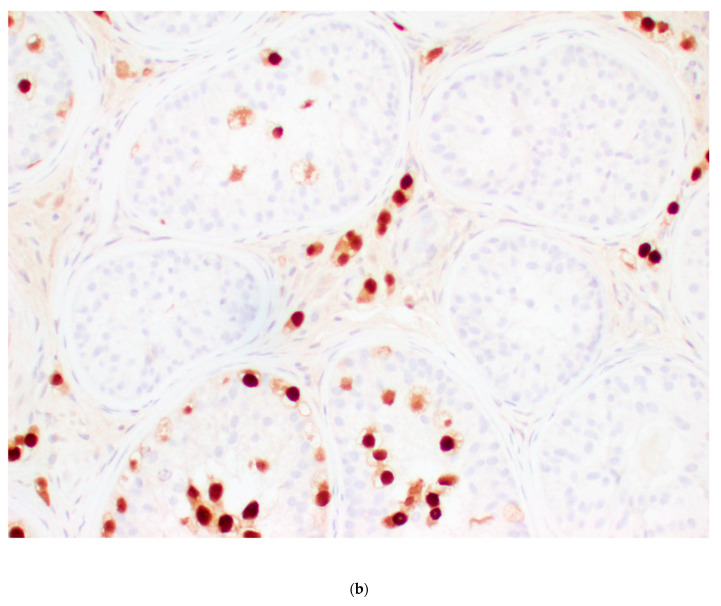

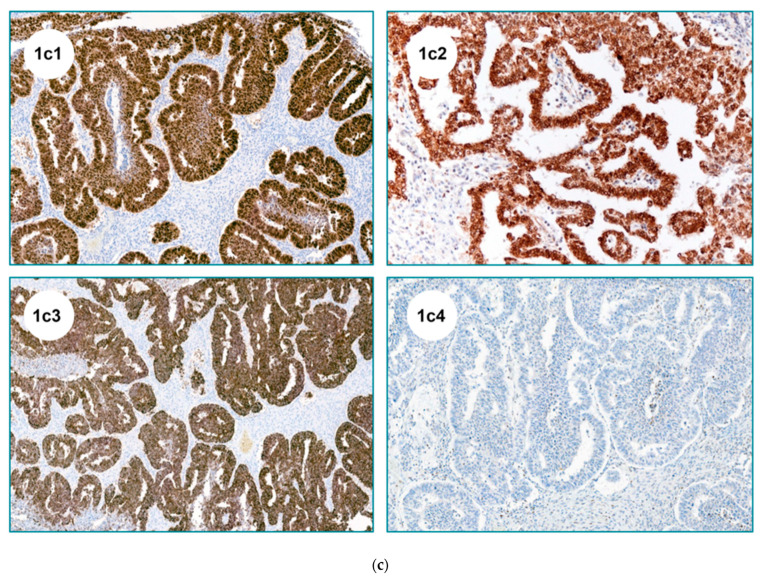
TGCT subtypes in the tumorigenesis of TGCT. (**a**). The epigenetic landscape for germ cells. The germ cells progress from toti-differentiated cells to unipotent cells, and a gene panel of *OCT4*, *SOX2*, *LIN28*, and *NANOG* (OSLN) can reprogram germ cells for induced pluri- to totipotent stem cells (iPSC). The red boxes show gene panels for iPSC. The progression of the TGCT subtypes implies a loss of chromosomes from tetraploid malignant germ cells over hypertriploid seminoma to hypotriploid embryonic carcinoma. (**b**). Microinvasive germ cell tumor (MGCT). MGCT is the first invasive step in the tumorigenesis of TGCT. MGCT is an intermediary precursor of TGCT that has malignant germ cells inside and outside seminiferous tubules in the testis. The figure shows MGCT stained for OCT4, the protein of *POU5F1*. (**c**). Embryonal carcinoma (EC). EC has a positive homogeneous immunohistochemical staining for three transcription factors for induced pluripotent stem cells (OCT4, SOX2, and NANOG) and a homogeneous negative staining for the protein of the tumor suppressor gene, *RB1*. EC stained immunohistochemically positive for OCT4 (1c1), SOX2 (1c2), LIN28 (1c3), and immunohistochemically negative for RB1 (1c4).

In 1957, Waddington described an epigenetic landscape of differentiation where a one-way road leads totipotent stem cells over pluripotent stem cells to unipotent differentiated cells (Figure 1a) [1]. Later studies show that gene panels are able to reprogram unipotent differentiated cells into induced pluripotent stem cells (iPSC) [2,3]. An iPSC panel includes *POU5F1* and *SOX2,* combined *with LIN28 and NANOG* (the Thomson OSLN panel) [2]. A second iPSC panel includes *POU5F1* and *SOX2,* combined with *KLF4* and *MYC* (the Yamanaka OSKM panel) [3]. iPSC can differentiate into teratoma (TER) [4]. The iPSC concept represents a major paradigm shift which points to a bi-directional two-way road for differentiation in the Waddington landscape.

Two precursor lesions for testicular germ cell tumors type II, germ cell neoplasia in situ (GCNIS) and microinvasive germ cell tumor (MGCT) (Figure 1b) [5,6], and seminoma (SE) also express an OSKM panel, with SOX17 as substitute for SOX2. In SE, *SOX17* stimulates genes that inhibit differentiation [7] but allows SE to be transformed into embryonal carcinoma (EC) [8]. EC is the undifferentiated nonseminomatous (NST) subtype of testicular germ cell tumors type II. The transformation includes a shift from a highly expressed SOX17 in SE to a highly expressed SOX2 in EC and a shift from the OSKM to the OSLN panel [9]. The iPSC panels are driver genes in the tumorigenesis of testicular germ cell tumors type II.

**Figure 2:**
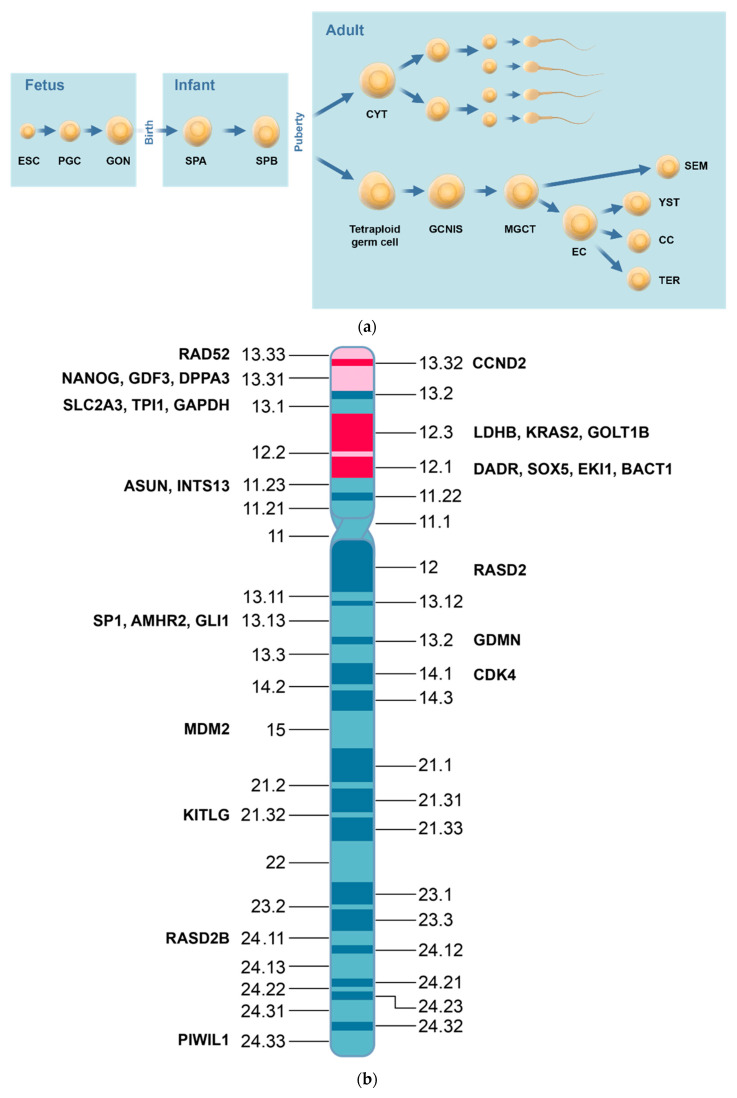

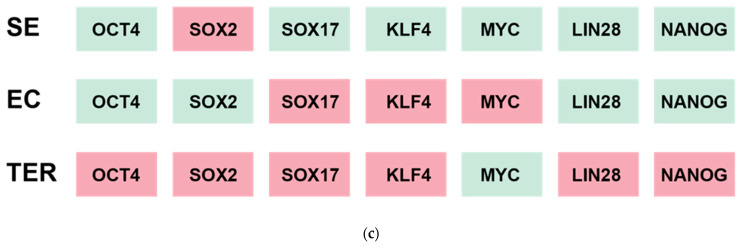
Development of normal and malignant testicular germ cells. (**a**) Shows the development of normal testicular germ cells from fetal life to spermatozoa in adulthood, and the development of malignant testicular germ cells from tetraploid malignant germ cells to the testicular germ cell tumors type II subtypes. Seminomas are diagnosed at a higher age than non-seminomatous testicular germ cell tumors as reflection of a lower proliferation rate in seminoma. (**b**). Chromosome 12. The short arm of chromosome 12 (12p) has two candidate regions that are especially important for the tumorigenesis of testicular germ cell tumors type II, shown in red. The candidate regions have gene loci for three driver genes: *CCND2, NANOG* and *LDHB*. The candidate regions are shown in red. The figure also shows other genes that literature and OMIM relate to TGCT. (**c**). IPSC gene panel in TGCT subtypes. The combined OSKM/OSLN panels for seminoma, embryonal carcinoma, and teratoma have dramatic differences with up- and down-regulations of the genes. Down-regulated genes are indicated with red and up-regulated genes are indicated with green.

EC highly expresses the OSLN panel (Figure 1c) [6]. EC can differentiate both into an embryonic subtype, TER, and into two extra-embryonic subtypes, yolk sac tumor (YST), and choriocarcinoma (CC) (Figure 2a) [6]. As EC differentiates into TER, the OSLN panel is silenced (Figure 1a). As proof of principle for the Waddington landscape has a two-way road of differentiation, Eldar-Geva et al. reported that fibroblasts from a 32-year-old SE patient were reprogrammed into iPSC [10]. A circuit of *OCT4, SOX2,* and *NANOG* is crucial for the tumorigenesis of testicular germ cell tumors type II [11]. One of the authors of this review (FFN) was the first to argue that iPSC is crucial for some patients with EC [12].

In this review of the tumorigenesis of TGCT, we examine whether DNA methylations and histone modifications modify the expression of driver genes between the subtypes of testicular germ cell tumors type II, and whether epigenetic writers, readers, and erasers modulate the modifiers.

## 2. Testicular Germ Cell Tumors Type II

### 2.1. Pathology

There are seven types of testicular germ cell tumors [13]. Testicular germ cell tumors type I are tumors in early infancy. Testicular germ cell tumors type II (denoted TGCT in our review) have two main groups where patients with SE are diagnosed at a median age of 35 years and patients with NST are diagnosed at a median age of 25 years. Type III consists of spermatocytic tumor (ST). Patients with ST had a median age > 50 years. Type IV and V are mainly female germ cell tumors. Type VI are germ cell tumors that arise due to reprogramming for iPSC.

TGCT is unique in oncology. Of the seven germ cell tumor types, only TGCT originates from GCNIS, first described by Wilms in 1896 [14]. In oncology, only EC develops into TER. TGCT has a pathognomonic genetic abnormality, an isochromosome of the short arm of chromosome 12, i (12p) [15,16,17,18,19]. TGCT has a unique network with downregulated *CDKN2A* (p16 (INK4), *CDNKN2C* (p18INK4C), *CDKN2D* (p19INK4D)), and *CDKN1A* (p21, CIP1, WAF1), upregulated *CCND2*, and downregulated *RB1* [20]. The genes synergistically inhibit suppression of proliferation.

Compared with other malignancies, patients with TGCT have a unique isoenzyme pattern for serum lactate dehydrogenase (S-LDH) with an unusually high LDH isoenzyme 1 activity (S-LDH-1) [21,22,23]. LDH-1 is a tetramer of four LDHB subunits, generated from *LDHB* on the short arm of chromosome 12, 12p [24]. Of all malignancies, the international tumor, nodes, and metastases (TNM) classification incorporates serum tumor markers only for TGCT [25]. The serum tumor markers are serum alpha fetoprotein (S-AFP), serum human chorionic gonadotropin (S-hCG), and S-LDH. The classification was adopted from a study by the International Germ Cell Consensus Classification Group [26]. Metastatic TGCT responds extraordinarily well to platin-based chemotherapy, so Einhorn called TGCT a model of a curable solid neoplasm [27]. 

For young adult Caucasian men, TGCT is the most frequent malignancy, and the incidence has increased worldwide in many recent decades [28,29,30]. 

The TGCT subtypes differ in frequency. For 78% of testes with TGCT, GCNIS is present in the testis besides the TGCT [31]. A Danish Testis Cancer (DATECA) group study reported a five-year nation-wide cohort of patients with TGCT where a central review reevaluated the TGCT histology according to a World Health Organization (WHO) classification [32]. The cohort included 1058 patients. Half of the patients had SE, slightly less than half of the patients had NST (including those with both SE and NST), and one percent of the patients had ST. Of the NST patients, three quarters of the patients had an EC component in the tumors, half the patients had a TER component, and less than a quarter of the patients had a YST component or a CC component. The patients were followed with regular monitoring of S-AFP and S-hCG [33]. The serum tumor markers were important for staging and outcome of treatment.

### 2.2. Tumorigenesis

As background for TGCT, endogenous androgen levels during puberty and early adulthood are inversely associated with the risk of TGCT [34]. Severe acne reduced the risk of TGCT with 50%. TGCT develops from normal germ cells through precancer lesions and subclinical precursor lesions to macroscopically overt tumors (Figure 2a) [35,36,37,38,39]. On ultrastructural level, testicular malignant germ cells resemble spermatogonia type A more than gonocytes [40].

### 2.3. Cytogenetics

The TGCT tumorigenesis is governed by cytogenetic abnormalities. To illustrate the cytogenetic abnormalities, long-term cultures of primordial germ cells gradually increase the copy number of chromosome 12, 17, and X, similar to the pattern in TGCT [41,42,43,44,45]. 

Walt et al. found that tetraploidization is the first step in the TGCT tumorigenesis [46]. Testicular germ cells have several ways to tetraploidization. Cells that are deficient in *CDKN1A* (p21/Cip1) can bypass a G2 block and conduct a second S phase before they undergo mitosis [47]. TGCT has deregulated *AURKA, AURKB*, and *AURKC*, that may lead to abnormal chromosomal segregation and cytogenesis during cell divisions [48,49]. Later, malignant tetraploid germ cells loose chromosomal material [37]. In consequence, SE consists of hypertriploid cells and NS of hypotriploid cells [36]. GCNIS and SE have a higher ploidy than NST [50,51]. 

Comparative genomic hybridization of TGCT shows the tumors have gains of parts of chromosome 7, 8, 12p, 14, and X, and losses of parts of chromosomes 3, 4, 5, 10, 11, 12q, 16, 18, 22, and Y [52]. 

Of the TGCT subtypes, only a minority of SE patients have *KIT, KRAS2*, and *NRAS* mutations [51]. SE has a rate of 0.06 mutations/Mb [53]. TGCT has a rate of 0.5 mutations/Mb whereas other malignancies have mutation rates of 4.0 mutations/Mb [54].

### 2.4. Genetics 

The TGCT subtypes differ in gene expression and the differences contribute to the TGCT tumorigenesis [55]. Two regions of chromosome 12p are important for the tumorigenesis (Figure 2b) as the regions have gene loci for *NANOG, CCND2, LDHB,* and *KRAS2*. DNA methylation increases in the progression from GCNIS to differentiated NST subtypes [56,57,58,59]. 

The gene expression of the OSLN/OSKM panels changes dramatically as SE develops into EC and TER (Figure 2c) [6]. Thus, *POU5F1/OCT4, SOX17, SOX2, KLF4, MYC, LIN28,* and *NANOG* are driver genes for TGCT. Other driver genes in undifferentiated TGCT subtypes are upregulated oncogenes *CCND2* [60], *KLF4* [61], and *MYC* [62] and a downregulated tumor suppressor gene *RB1* [63]. 

Many candidate genes for TGCT have their gene locus on chromosomes other than the short arm of chromosome 12 (12p). *AFP* has gene locus on 4q13.3, *TET2* on chromosome 4q24, *SOX17* on 8q11.23, *MYC* on 8q14.13, *POU5F1* on 8q24,21, *KLF4* on 9q31.2, *RB1* on 13q14.2, *CCGB3* on 19q13.33, and *DNMT3B* on 20q11.1. Similarities and differences in expression of the driver genes between the TGCT subtypes form a biologic chain for the TGCT tumorigenesis. 

The OSLN/OSK panels change dramatically in expression as SE progresses to EC and TER (Figure 2b). Environmental factors regulate gene expressions in TGCT through a KIT/KITL signal transduction pathway [64]. 

Normal germ cells express PRAME and *LDHC,* the gene for the subunit of a testis specific lactate dehydrogenase isoenzyme, LDH-C [65,66,67], but not *KIT* and *NANOG*. Normal germ cells, GCNIS, and SE express PRAME but not NST [68,69,70]. GCNIS expresses *PRAME, KIT*, and *NANOG* but not *LDHC*. Normal male germ cells express *RBMY1A,* whereas the gene is silenced in SE and EC [71].

GCNIS and embryonic stem cells have similar gene expressions [72,73,74]. In GCNIS, *POU5F1, NANOG, SOX17*, *LIN28,* and *KIT* were among the twenty genes with the highest gene expression. Invasion as GCNIS progresses to MGCT is associated with i (12p) [75,76], downregulated *CDKN1A* (p21), and upregulated *MDM2* [77]. SE highly expresses *POU5F1* [78,79,80]. Microenvironment factors such as TGF-beta, EGF, and FGF4 support the transition from TCam-2 to EC [81].

The development from the SE expression of *SOX17* to the EC expression of *SOX2* is part of the iPSC changes between the TGCT subtypes (Figure 2c) [82]. The changes show TGCT has a cellular plasticity. SE has OCT4 and SOX17 as partners, and EC has OCT4 and SOX2 as partners [7]. *NANOG* is not expressed in normal testicular germ cells, highly expressed in SE and EC, and silenced in YST, CC, and TER. Moreover, SE and EC overexpress *POU5F1* and *NANOG* whereas TER does not [82].

EC and YST highly express the NODAL co-receptor *CRIPTO* and GCNIS, SE, and CC have a lower expression [83]. Immunohistochemistry links AFP in NST in patients to S-AFP [84] and links hCG in TGCT in patients to S-hCG [85]. 

Retinoic acid stimulates EC to differentiate into TER. The differentiation is associated with downregulated *POU5F1* leading to downregulated *PUMA* and *NOXA* and reduced apoptosis [86]. 

## 3. Epigenetics and TGCT 

### 3.1. Tumorigenesis

The TGCT tumorigenesis is also governed by epigenetics. Epigenetic regulation of genes in iPSC panels is important for the similarity between embryonic stem cells (ESC) and EC [87]. Normal germ cell tissue and TGCT subtypes differ in epigenetics [57]. Normal tissues have imprinted genes and methylated LINE1 and Alu elements in the DNA string whereas both SE and NST have unmethylated LINE1. SE has unmethylated Alu elements whereas NST has methylated Alu elements. TGCT expresses *TET1* and the thymine DNA glycosylase *TDG* supporting that TGCT demethylate methylated DNA using an oxidative pathway [88]. MiR-223-3p regulates TGCT growth and apoptosis [89].

Of the TGCT precursors, GCNIS expresses *DNMT1* and has generally hypomethylated DNA. GCNIS had elevated H2A.Z, mono-, di-, and trimethylated histone 3 lysine 4 (H3K4me1, H3K4me2, and H3K4me3) and H3K9me2, and acetylated histone 3 lysine 9 (H3K9ac), and low H3Kme2 and H3K29me2 [90,91]. In GCNIS, hypomethylation is not solely due to lack of demethylation. Regarding demethylation, GCNIS does not have the methylcytosine dioxygenases *TET1* and *TET2*, so GCNIS uses an AID/APOBEC1 pathway for the demethylation [90]. 

Of TGCT subtypes, SE had elevated H3K4me1 and H3K9me2 and low H3K4me2, H3K4me3, H3K9ac, and H3K27me3. In SE, an OCT4/SOX17 complex binds to a compressed motif CTTTGTATAAAT [92]. The *SOX2* promoter has H3K27me3 and polycomb complex 2, and suppresses *SOX2* [93]. Further in SE, the compressed motif together with other OSKM genes hinder differentiation of SE and gives SE a poised pluripotency. In malignant germ cells, the canonical motif is located in enhancers of genes for pluripotency whereas the compressed motif is located in regulatory regions of genes for differentiation into endodermal structures. TGCT expresses *HDAC1, HDAC2,* and *HDAC3*, where CC has a high *HDAC2* [94]. 

TCam-2, a SE cell type, does not express SOX2 [93,95]. TCam-2 has elevated H3K4me3 and H3K27ac for *SOX17* and low H3K4me3 and H3K27ac for *SOX2* whereas NCCIT, an EC cell line derived from a mediastinal germ cell tumor, has elevated H3K4me3 and H3K27ac for *SOX2* and low H3K4me2 and H3K27ac for *SOX17* [96,97]. TCam-2 expressed *SOX17* more than NCCIT. In TCam-2, elimination of an upstream region of *SOX17* downregulated *POU5F1, NANOG*, and *LIN28* [7]. 

TCam-2 xenotransplanted to the abdominal flank in mice upregulate *NODAL* to express EC genes [97]. The upregulation of *NODAL* was mediated through a hypomethylated *DNMT3B, GDF3, DPPA3, SOX2, LIN28,* and *ZIC3*. 

SE had <1% methylations of CpG/CpH sites whereas EC had frequent CpH methylations [51]. Comparing SE and EC, *DNMT3B* and *TET2* were the genes that differed most in expression [98]. EC has moderate levels of H3K4me2 and H3K4me3 and no H3K9me2 and H3K27me3. 

In EC, an OCT4/SOX2 complex binds to a canonical motif CTTTGTCATGCAAT [7,92]. It is located in enhancers of pluripotency genes whereas the compressed motif is located in regulatory regions of genes for differentiation into endoderm structures. 

In TCam-2, SOX17 bound more to the compressed motif of OCT4 and SOX2 than to the canonical motif whereas in 2012p, a NST cell type, SOX2 bound equally well to the canonical and compressed motifs. The canonical motif activates *GDF3, POU5F1, SALLA, SOX2*, and *TP53.*

In P16 cells, a NST cell type, silencing of *OCT4, KLF4, MYC*, and *NANOG* impaired tumor growth [99]. 

Additionally, methylations of H4 arginine can actively regulate expression of genes [100,101]. 

### 3.2. DNA Methylations

The 5-C methylation of cytosines in the DNA string changes the chromatin structure in DNA from heterochromatin to euchromatin and opens it for gene expression (Supplementary Figure S1). The level of DNA methylation increases as GCNIS progresses through undifferentiated TGCT into differentiated NST. Comparing the TGCT subtypes, the expression of TET was inversely associated with DNA methylation [82]. GCNIS and SE did not stain for 5-^m^C, whereas EC had a moderate staining, and YST, CC, TER, drug-resistant SE, TCam-2, and NS cell lines NT2, 2012p, and NCCIT stained homogenously positive [102]. In TGCT, a methylated gene body increases expression of the gene whereas a methylated promoter of the gene inhibits expression of the gene [103].

GCNIS expresses both *DNMT1, DNMT3A,* and *DNMT3B* [90]. For restitution, GCNIS expresses a base excision repair (BER) protein that makes cells reestablish cytosines at abasic sites where the cytosines previously had been eliminated [104]. GCNIS has an extremely low level of 5-^m^C [20]. 

SE overexpresses *TET1,* has low proportions of 5-^m^C and 5-^mh^C, and no CpG methylations at CpG sites [82]. SE upregulated *TET1, TET2,* and *TET3* [82,88]. SE and EC had median 17% and 31% complete methylations of CpG sites in the upstream region for *POU5F1*, whereas YST and TER had the methylations in three quarters of the CpG sites [105]. SE and differentiated NST expressed *MYC,* but differed in gene networks and miRNA [106].

TCam-2 expressed *TET2* more than the NST cell lines NT2, 2012p, and NCCIT [98]. Compared with SE, EC had significantly more methylations of promoters of the tumor suppressor genes *MCAM (p <* 0.0005, chi2 test), *MGMT (p <* 0.0005), *MLH1 (p =* 0.004), *S100 (p <* 0.0005), *VGF (p =* 0.011, and *FKBP4 (p <* 0.0005) [107]. EC expresses *DNMT3B* and *EHMT2* more than SE [105]. EC has hypermethylated promoters in 40 downregulated genes [71]. Transfection of micro-RNA 630 into a NT2/D2 EC cell line targeting the 3′UTR of *POU5F1, SOX2,* and *NANOG* inhibited the expression of the genes [108]. TGCT has methylated repetitive elements in the DNA strand [57]. Differentiated NST had CpG methylation similar to solid cancers [82]. 

### 3.3. Histone Modifications and Modulations

Methylations and acetylations of histone 3 lysines change DNA structures from hetrochromatin to euchromatin and opens them for gene expression (Supplementary Figure S2a,b). Histone modifications also contribute to the TGCT tumorigenesis. In normal germ cells, upregulation of E2F1 in the CCND2/RB1 signal transduction pathway gave rise to atypical mitosis and cell atypia like that in GCNIS [109]. 

**Figure 3:**
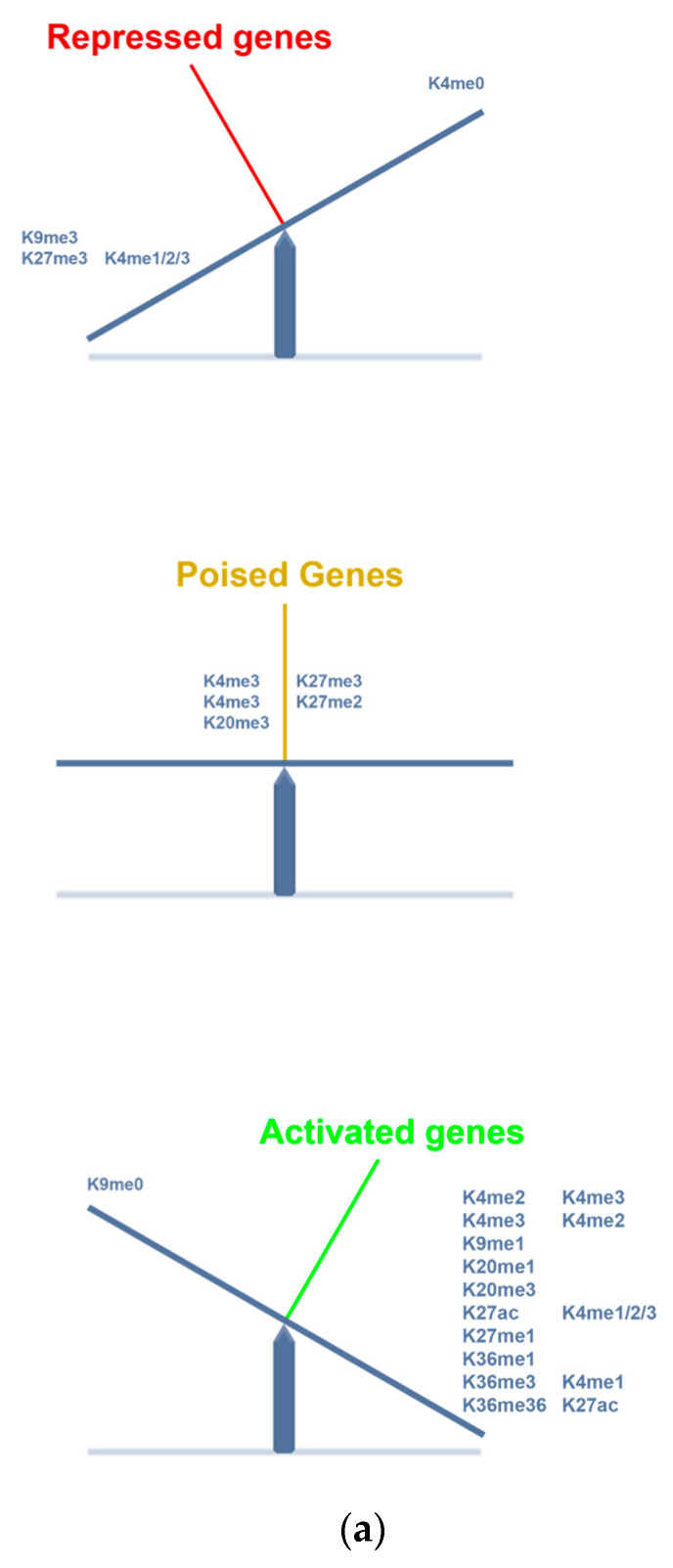

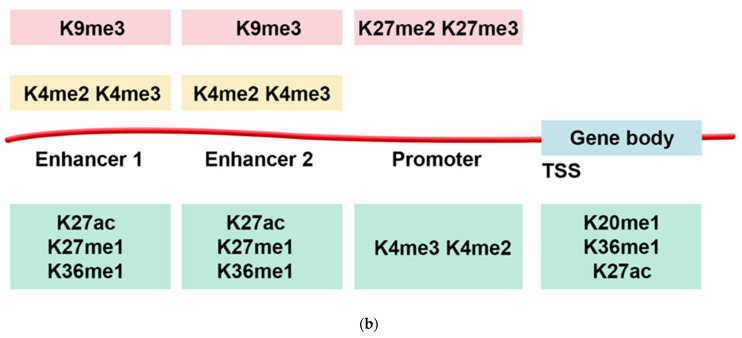
Epigenetic regulation of gene expression. (**a**). Histone modifications and gene expression. Histone lysine methylations and acetylations can lead to repressed, poised or activated gene expressions. A combination of histone marks that act both to repress and activate a gene leads to a poised regulation of expression of the gene. (**b**)**.** Sites for histone marks and gene regulation. Methylated histone 3 lysines differ in action sites on enhancers, promoters, and gene bodies of genes. Red indicates down-regulated gene expression and green indicates upregulated expression of the gene.

EC had H3K4 and H3K9 methylations [110]. EZH2, *e*nhancer of Zeste-2, is a histone methyltransferase (“writer”) and a catalytic subunit in the polycomb repressor complex 2 (PRC2). EZH2 methylates H3K27, condenses chromatin structure, and silences tumor suppressor genes *CDKN2A* (p16(INK4)) and *RB1* (Figure 3a). Silencing *EZH2* with siRNA reduced cellular DNA replication and cell growth. A lysine specific demethylase 1A, KDM1A/LSD1, demethylates H3K4me2 [111].

H3K modifications differ by their binding sites on the genes as they regulate expression of genes. H3K4me3 and H3K27ac activate enhancers whereas H3K4me1 poises enhancers (Figure 3a). Akt-phosphorylated OCT4 stabilized OCT4, facilitated its nuclear localization, and its interaction with SOX2 [112]. 

Normal testicular tissue highly expresses *EZH2* whereas the expression is lower in GCNIS, and further reduced in invasive TGCT [113]. In EC cells, a H3K36 methyltransferase *SETD2* trimethylated H3K9, H3K27, and H3K36 on sites in *OCT4, SOX2*, and *NANOG* [114]. In 2102p, knockdown of *DNMTB* increased H3K27me3, EZH2 expression, and sensitivity to cisplatin [114]. TGCT has an antagonism between the regulation with H3K4me3 and H3K27me3. The antagonism gives TGCT a cellular plasticity and supports pluripotency. In *POU5F1*- and *SOX2*-positive EC cells, inhibition of LSD1 reduced proliferation whereas LSD1 inhibition of *POU5F1*- and *SOX2*-negative EC cells did not change the proliferation [111]. 

TER moderately expresses H3K4me2, H3K4me3, H3K9me1, H4K9me2, and H3K9me3, whereas YST strongly express the histone marks.

Combined, H3K4me3 and H3K27me3 in promoters of genes generate poised/bivalent signals for expression of genes [115].

### 3.4. Driver Genes

Epigenetics regulate driver genes in TGCT (Table 1). TCam-2 have low levels of H3K4me3 and H3K27ac whereas NST cell lines have high levels [96]. The driver genes *POU5F1, SOX2, LIN28*, and *NANOG* form a network of genes [116]. Upstream of the gene body, *POU5F1* has four conserved regions that can bind SOX2, NANOG, and HIF2A (*HIF-2ꬰ, EPAS1*) [117]. Dimethylated promoter for *POU5F1* increased expression of *POU5F1*, whereas reduced methylation reduced expression of the gene [110]. OCT4 binds to the ATTTTGCAT motif.

*POU5F1* is expressed in most GCNIS and all SE and EC [118]. NST cell lines have a network between *DNMT3B* and *POU5F1* [119]. Many transcription factors may be involved with the SOX2-OCT4 connection.

TCam-2 has low H3K4me3 and H3K27ac whereas NST cell lines have elevated levels. In EC cells, overexpression of *POU5F1* inhibited the expression of *NANOG* but not the expression of *FGF4* and *UTF1* [120]. In malignant germ cells, ACT-driven phosphorylation of OCT4 stabilizes OCT4, stimulates its interaction with SOX2, and increases cell survival [112]. Many transcription factors may be involved in the SOX2-OCT4 connection. In EC cell lines, LSD1 inhibition downregulated OCT4 and SOX2 [121].

OCT4-activating compounds (OAC1) activate genes stimulated by *POU5F1* and *NANOG* [122]. 

In NCCIT cells, siRNA downregulation of *POU5F1* reduced expression of *SOX2, LIN28,* and *NANOG,* and upregulated genes for differentiation, such as *OTX1, HAND1*, and *LAMB1* [123]. In EC cell lines, *miRNA-27* inhibited expression of *POU5F1* and made the cells differentiate [124]. Downregulation of *OCT4* increased miR-27, and the increase of miR-27 down-regulated *NANOG* [125]. Treatment of an EC cell line with an LSD1 inhibitor downregulated *OCT4* and *SOX2* [121].

SOX17 is hypomethylated in EC [126]. Deletion of SOX17 reduced expression of *POU5F1* [7]. In malignant germ cells, the switch from *SOX17* to *SOX2* determines whether cells function as SE or as EC [127].

Deletion of SOX17 reduced expression of *POU5F1*. TCam-2 had an elevated H3K4me1 in SOX17 whereas NCCIT had a low level [128]. Methylation of the *SOX17* promoter downregulated expression of *SOX17* [129]. The *SOX17* promoter has 420 base pairs and 48 GpC sites [130].

SOX2 is suppressed in SE by the polycomb repressive complex and H3K27me3 [93]. SE and EC has a hypomethylated *SOX2* whereas *SOX2* in TER has a higher methylation [131]. TCam-2 needs expression of *SOX2* for being reprogrammed into EC [7].

NST cell lines have a network between *DNMT3B* and *POU5F1*.

EC expressed *SOX2* three times more than TCam-2. In NCCIT cultures, silencing of *SOX2* gave prominent cell death within three days [123].

*LIN28* has a role in the TGCT tumorigenesis [132]. A distal enhancer of *LIN28* has three binding sites for OCT4 [133]. *LIN28* mediates effects by downregulated LET-7 microRNA [134]. SiRNA downregulated *LIN28* and reduced expression of *SOX2* and *NANOG*. In P19 malignant germ cells, silencing of *LIN28* reduced proliferation and tumor formation [132].

Regarding *NANOG*, Nettersheim et al. denoted a region upstream the transcription start site the NANOG regulatory region (NRR) [135]. It has binding elements for OCT4 and SOX2 [136,137]. Binding of the transcription factors to NRR increases the expression of *NANOG* whereas methylation of NRR inhibits the transcription factors which are bound to NRR and inhibits the expression of *NANOG*. GCNIS, SE, and EC express *NANOG* whereas TER does not [138]. TGCT cell lines with unmethylated NRR express *NANOG* whereas cell lines with methylated NRR do not. In EC cells, two transactivating domains in the C-terminal region of NANOG mediate expression of *NANOG* [139].

### 3.5. Epidrugs and TGCT

Drugs that inhibit epigenetic regulation of gene expression are denoted epidrugs. They have been studied in TGCT (Table 2). In a cohort study of treatment with the methyltransferase (“writer”) inhibitor 5-azacytidine (5-aza), 2 of 4 TGCT patients had partial remission [140]. Another study reported that one of 17 patients with TGCT treated with 5-aza had no evidence of disease at the end of follow-up [141]. In TCam-2, 5-aza increased expression of *POU5F1* and *NANOG*. Trichostatin works synergistically with retinoic acid [142].

EORTC conducted a trial of NST patients treated with the DNA methylation inhibitor 5-aza-2′-deoxycytidine (5-aza-dC) [145]. The response to 5-aza-dC depended on the cellular expression of *POU5F1* and *DNMT3B*. Treatment with 5-aza-dC increased the expression of *DNMT3B*. For patients with cisplatin-resistant metastatic TGCT, treatment with cisplatin and an inhibitor of DNA methyltransferase, guadecitabine, did not give long-lasting complete remissions [143].

Epidrugs for TGCT have mainly been evaluated in cell cultures of malignant germ cell lines because most patients with metastatic TGCT are cured with chemotherapy. In the TGCT cell lines NCCIT and 2012p, 5-aza had long-lasting effects [144]. TGCT cell lines have an inverse relation between sensitivity to 5-aza and sensitivity to cisplatin [115]. In EC cells, sensitivity to 5-aza demanded a highly expressed *DNMT3B* [146,147]. 

In 1994, Jutterman et al. were the first to report treatment with 5-aza-dC [148]. They found that 5-aza stimulated TCam-2 further and upregulated *POU5F1* and *NANOG*. Three EC cell lines, including Tera-2, were treated with 5-aza [149], and 5-aza-dC demethylated the promoter of *POU5F1* and reduced expression of *POU5F1*. Treatment with low dose 5-aza-dC demethylated promoters for *SOX2, NANOG*, and *MYC* [146]. In two NS cell lines, NCCIT and Tera-2, treatment with 5-aza-dC reduced H3K4me2 in the promoter of *POU5F1* and inhibited expression of *POU5F1*. 

Regarding inhibitors of histone 3 modifications, treatment of an ovarian germ cell tumor cell line PA-1 with an inhibitor of the histone methylase LSD1, CBB3001, downregulated OCT4 and SOX2 and inhibited tumor growth [121].

Treatment of the EC cell line P19 with a histone lysine demethylase (KDM) inhibitor, tranylcypromine, inhibited a histone demethylase, increased H3K4 methylation, and expression of *POU5F1* [150]. In addition, a chimeric inhibitor animacroxam was effective [151]. 

In cisplatin-sensitive and cisplatin-resistant EC cell lines, two inhibitors of histone lysine deacetylase (HCAD), belinostat and panobiostat, were effective [152]. In NST cell lines, also the HDAC inhibitor depsipeptide was effective [153]. A bromodomain inhibitor, JQ1, binds to the amino-terminal twin of bromodomains of BET proteins that bind to acetylated histone lysines. In an EC cell line, JQ1 downregulated pluripotency factors and caused the cells to differentiate into mesodermal structures [154]. 

Treatment with the HDAC1 inhibitor, romidepsin, increased H3K acetylations and decreased ARI1A and thereby expression of *POU5F1, SOX2, LIN28,* and *NANOG* [155]. Treatment of the EC cell line P19 with the HDAC inhibitor trichostatin (TSA) inhibits cell progression [121]. A study compared treatment of germ cell tumor cell lines with seven epidrugs [156]. 

The CDK inhibitor YKL-5-124 inhibited both TCam-2 and 2102p whereas SY0351 and NVP2 gave cell line-specific responses [157].

## 4. iPSC in Oncology

### 4.1. TGCT 

We confirmed and expanded the role iPSC has for the tumorigenesis of TGCT based on epigenetic regulations of driver genes, not least genes in iPSC panels. Epigenetic mechanisms regulate the shift from an OSKM-like panel in SE to an OSLN panel in EC, and the silencing of the OSLN panel in TER. iPSC genes form a biologic background for the genetic and histologic changes between the TGCT subtypes. DNA methylations and histone modifications regulate iPSC genes. Driver genes reprogram malignant germ cells to pluripotency, increase unlimited self-renewal and independency of growth factors, and inhibit the suppression of proliferation, all hallmarks of malignancy [158].

In 1946, Friedman and Moore were first to point out that EC has a unique toti-differentiation potential which is crucial for the TGCT tumorigenesis [159]. In 1980, Pugh and Parkinson commented on the tumorigenesis and classification of TGCT and noted that clinicians generally lump the TGCT subtypes as SE and NS whereas pathologists generally split TGCT in subtypes [160]. Our review categorizes all TGCT subtypes as a continuum of progression. Correspondingly, oncologists generally treat patients with stage 1 SE and NST with active surveillance, and treat patients with metastatic SE and NS with BEP chemotherapy.

The evidence regarding the OSKM and OSLN panels motivates an iPSC hypothesis for the TGCT tumorigenesis. The first premise is that iPSC panels can reprogram cells to iPSC and that only iPSC and EC can differentiate into TER. A second premise is that OSKM and OSLN panels can reprogram both normal somatic cells and malignant germ cells into an iPSC/induced totipotent stem cell pattern. Thus, iPSC panels can reprogram cells with normal and abnormal ploidy into iPSC/induced toti-potential cells. 

A third premise is that EC expresses the OSLN panel and is able to differentiate into YST, CC, and TER whereas normal somatic cells reprogrammed with the OSKM panel to iPSC only are able to differentiate into TER. Accordingly, the cellular background before reprogramming for iPSC determines the frames for differentiation of IPSC/toti-potential cells.

GCNIS develops into MGCT within median five years [161]. The median age at diagnosis for SE patients is 35 years and 25 years for NST patients, so it is more likely that thetumorigenesis of TGCT starts during puberty than during fetal life.

The regional site for xenotransplants of TCam-2 to mice is crucial for progression to EC [8]. The fact points out that the microenvironment governs expression of genes in iPSC panels, mediated by epigenetic mechanisms. Thus our review add an epigenetic dimension to the “genvironmental” hypothesis for the TGCT tumorigenesis proposed by Looijenga [162]. 

Many observations support the progression from SE to EC. An autopsy series of patients with primary SE showed that 63 (44%) of 145 patients had NST metastases at the time of death [163].

Epigenetics have a key role for the TGCT tumorigenesis, and gene mutations have a limited role. The Genomic Cancer Atlas (TGCA) study of patients with TGCT reported that of TGCT subtypes, only SE had mutations in *KIT, KRAS*, and *NRAS* [51]. The shifts in gene expression associated with progression between the TGCT subtypes exclude that mutations in general regulate the expression of the driver genes and the progression of the TGCT subtypes.

The iPSC panels also govern clinical characteristics between the TGCT subtypes. The low *SOX2* expression in SE and high *SOX2* expression in EC explain why SE mostly is the only TGCT subtype in SE tumors, and why NST TGCT most often incorporates more than one NST subtypes. The transition from SE to EC explains why TGCT often combine SE and NST elements.

The pattern of gene expression also explains why SE proliferates more slowly than EC [164], and why patients with SE have a median age at diagnosis that is ten years higher than that for patients with NST. Furthermore, the switch from SOX17 to SOX2 between the TGCT subtypes explains why SE responds better to platin-based chemotherapy than EC, and why SE is sensitive to radiation therapy in contrast to the radiation-insensitivity of NST subtypes [165]. As EC differentiates into TER, the switch in expression of iPSC genes contributes to the cisplatin-resistance in TER.

Our review includes three driver genes with gene locus on chromosome 12. In addition, reprogramming of TCam-2 to EC also upregulated three genes *DPPA3, GDF3*, and *BCAT1* [97], that also have their gene locus on chromosome 12p. 

Several gene networks collaborate in the TGCT tumorigenesis. We report that IPSC genes work synergistically in a network. A second network is the CCND2/RB1 signal transduction pathway, and a third network is genes on the short arm of chromosome 12 associated with anaerobic glycolysis [166]. *POU5F1* also participates in a fourth network that links macro-environmental factors such as retinoic acid and cisplatin to a downregulated *POU5F1*, causing downregulated *NOXA* and *PUMA,* and reduced apoptosis [86]. 

The link between i (12p) and extratubular invasion of malignant germ cells implies that *CCND2, NANOG*, and *LDHB* (with gene loci on chromosome 12p) are more important for the progression between the TGCT subtypes than *KITL* and *CDK4* (with gene loci on chromosome 12q).

National and international studies support that S-AFP, S-hCG, and S-LDH are prognostic indicators for TGCT. One of the authors (FEvE) was first to report the prognostic value of S-LDH in 1978, and two multivariate analyses later confirmed the prognostic value [167,168,169]. The prognostic value of S-LDH was accepted worldwide after the international large collaborative study in 1997 [26]. Recently, the Global Germ Cell Tumor Collaboration documented the prognostic value of S-LDH for patients with advanced SE (hazard ratio 2.9, *p* = 0.003) and patients with NS with intermediate prognosis (hazard ratio 2.6, *p* < 0.001) [170]. 

As TGCT suppresses *LDHC,* patients with TGCT and elevated S-LDH do not have detectable S-LDH isoenzyme C [171]. Of present serum tumor markers, only S-LDH-1 is generated from a gene with locus on the short arm of chromosome 12 [172,173,174,175].

DATECA studies showed that patients with SE and NST stage 1 had similar good outcomes whether they were treated with radical orchiectomy followed with active surveillance or with adjuvant radiation therapy to pelvic and retroperitoneal lymph nodes [176,177]. Complementarily, DATECA publication of SE and NST TGCT stage 1 followed with surveillance reported that half of the patients had a raised S-LDH-1 before orchiectomy [178,179]. For the NS TGCT patients, a raised S-LDH-1 was associated with a reduced relapse-free survival (*p* = 0.003, log-rank test).

Clinically, most patients with metastatic TGCT are cured with a combination of chemotherapy regimen with bleomycin, etoposide, and (cis)platin (denoted BEP) [180]. In addition, some patients are cured with surgery for post-chemotherapy residual TER lesions. Some patients who do not achieve a complete remission with four courses of BEP can be cured with high-dose cisplatin-based chemotherapy [181]. Impressively in recent years, the good five-year overall survival for patient with TGCT improved further [182,183]. The improvement is due to the shift in combination chemotherapy from vinblastine to etoposide in the BEP regimen. For patients with high-risk metastatic TGCT who fail on standard BEP chemotherapy, the decay of two serum tumor markers, S-AFP and S-hCG, pointed to the cisplatin-resistance as early as after the first course of BEP [29,184]. Overall, the prognostic value of the three serum tumor markers is documented in prospective follow-up studies of more than 10,000 patients with TGCT [26,170,182,183].

It is a challenge that patients with metastatic TGCT given a high cumulative dose of cisplatin (>635 mg) have a mortality that increases dramatically after follow-up for ten to twenty years [185]. Accordingly, oncologists might consider to study new systemic treatments as alternative to high-cisplatin-dose chemotherapy.

### 4.2. Other Malignancies

The driver genes have broad implications in oncology. Small cell lung cancer (SCLC) and small cell esophageal cancer (SCEC) have elevated *SOX2* and low *RB1* and the two genes reduce inhibition of proliferation [186,187,188]. Synergy between the two genes explains why SCLC, SCEC, and TGCT grow faster than most other malignancies. Other malignancies also highly express the iPSC panels [189]. iPSC panels, especially *SOX2* and the OSN panel, are highly expressed in cancer stem cells of other malignancies [189,190,191,192]. 

*POU5F1, SOX2*, and *NANOG* are important for the tumorigenesis of skin, oral squamous, esophageal, lung, and colon cancer [193,194]. *SOX2* contributes to the development of brain tumors [195]. *NANOG* is also an important mediator of cancer induction [196]. For prostate cancer, high expression of *SOX2* reduces expression of *RB1* and *TP53* and changes the cancer from a luminal androgen-receptor-dependent subtype to a basal androgen-receptor-independent subtype [187]. Based on the morphology in prostate cancer, a Gleason score contributes to treatment decisions and predicts outcome of treatment [197]. The increase in Gleason score is denoted as dedifferentiation. 

SCLC downregulates both *RB1* and *TP53* whereas TGCT only downregulates *RB1*. The difference contributes to different outcomes where most patients with metastatic TGCT are cured with BEP chemotherapy whereas most patients with metastatic SCLC-who despite temporary response to EP-based chemotherapy-progress and die of the cancer. 

So far, the United States of America Federal Drug Administration (FDA) has approved seven epidrugs such as 5-aza, 5-aza-cD, belinostat, paninostat, PXD-101, romidepsin, and vorinostat as routine treatment for cutaneous T cell lymphoma, chronic myelomonocytic leukemia (CMML), and multiple myeloma, respectively (Table 2) [198,199,200,201,202,203,204]. A phase III trial of patients with myelodysplastic syndrome showed that 5-aza increased overall survival substantially (hazard ratio 0.58, *p* = 0.0001, log-rank test) [205]. The FDA also approved a CDK2/CDK6 inhibitor trilaciclib for patients with advanced SCLC treated with EP-containing regimens [206].

In contrast to TGCT, some other malignancies have mutation-based increases in gene expression that are more important than non-mutational increases. Patients with non-small cell lung cancer (NSCLC) with mutated epidermal growth factor receptor (EGFR) respond better to targeted systemic treatment with EGFR tyrosine kinase inhibitors (EGFR-TKI) than patients with a high non-mutational expression of EGFR [207]. Today, the best treatments of patients with NSCLC with activating EGFR mutations are third-generation EGFR TKI rosimertinib and a combination of first-generation EGFR TKI and chemotherapy [208].

## 5. Perspectives and Conclusions

Continued research may further expand the knowledge of epigenetic regulation of driver genes in oncology and of the TGCT tumorigenesis. In reprogramming cells for iPSC, other genes can substitute for the genes we report in our review [209,210]. These genes may also be relevant in TGCT tumorigenesis. Thus, the number of relevant driver genes in oncology and in the TGCT tumorigenesis may increase in future. New insight in regulation of TGCT driver genes may also be relevant for other malignancies. 

Innovative studies of driver genes in many malignancies have general oncologic relevance. For women with hormone-sensitive breast carcinoma, present epidrugs enhance effects of hormone treatment but also increase adverse effects [211]. The findings motivate new epidrugs with fewer adverse effects.

Investigations of epidrugs in TGCT cell lines have been inspired by the clinical achievements with epidrugs in other malignancies. Recent trials support combinations of epidrugs. For elder patients with acute myeloid leukemia, treatment with a combination of 5-aza and the BCL2 inhibitor venetoclax gave a better overall survival than monotherapy with only 5-aza [212]. Another study showed a synergy between a DNMT inhibitor and a HDAC inhibitor [213].

In addition to serum tumor markers, liquid biopsies monitoring hypermethylated driver genes may improve follow-up routines of patients with TGCT and other malignancies [214]. 

In TGCT, downregulated tumor suppressor gene *RASSF1A* increases proliferation [110,215]. TGCT also has a downregulated *RB1* [216]. It remains to be shown whether the two tumor suppressor genes act in synergy. A recent review pointed to three genes with gene locus on the short arm of chromosome 12, *GLUT3, GAPDH,* and *TPII*, that participate in anaerobic glycolysis [166]. The question remains whether *LDHB* and the three genes collaborate for a Warburg effect in TGCT [217,218]. 

**Figure 4:**
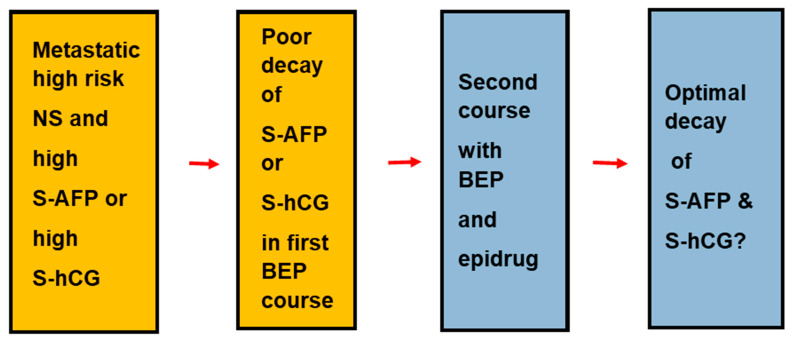
A new trial of epidrugs in TGCT. Candidate patients are patients with high-risk NS metastatic TGCT with insufficient decay of serum tumor markers after the first course of BEP chemotherapy. The proposed trial investigates whether combination of epidrugs and BEP chemotherapy might give response equal to high-dose salvage chemotherapy. The new elements in the trial are shown in grey boxes. If a patient fails to present with sufficient decay of two serum tumor markers, the patient will be switched to a routine high-dose cisplatin salvage chemotherapy.

For patients with TGCT, the target and timing may increase the efficacy of epidrugs. New epidrugs might specifically target the motifs of the combinations of OCT4 and SOX2/SOX17. As for more upfront timing, new trials may recruit patients with high-risk metastatic NS and limited decay of S-AFP and S-hCG after the first course of BEP. Such trials can investigate the efficacy of BEP combined with relevant candidate drugs as alternative to high-dose cisplatin chemotherapy (Figure 4).

In conclusion, genes that reprogram for iPSC have a major role in the TGCT tumorigenesis, and contribute to the aggressiveness in many other malignancies. 

## Figures and Tables

**Table 1:** Histologic types of TGCT and epigenetic regulation.

Expressed Transcription Factors	Histologic Types		NGC	GCNIS	SE	EC	TER
Epigenetic mediators		High	PRAME LDHC	PRAME, KIT, SOX17, NANOG	PRAME, KIT, SOX17, KLF4 POU5F1, NANOG, MYC	NANOG, POU5F1, DNMT3B	RB1, MYC
		Low		RB1	RB1	RB1, MYC	POU5F1, SO)X2, LIN28, NANOG
5mC			+++	Neg	Neg/++	+++	
5mhC				Neg	Neg	+++	
Epigenetic modifiers	H2A.Z		+/+++	+++	Neg/+	Neg/+	
	H3K4		Me1 +++ Me2/me3 ++	Me1/me2/me3 +++	Me1 ++/+++	Me1 neg/+ Me2/Me3++/+++	
	H3K9me1/me2			Neg/+	++	++	
	H3K9ac			+++	Neg/+	Neg/+	
	H3K27me2						
	H3K27me3		+++	Ac/+	+++	neg	
Epigenetic modulators	DNMT				Low	High	
	TET			Low	High	low	
HMT	EZD2			+++	Neg/+	neg	
HDMT	UTX		Neg	Neg	Neg	Neg	
	JMJDD3		Neg/+	Neg	Neg/+	Neg/+	
	HAT				High	Low	
	HDAC				Low	High	

The signs +/++/+++ indicats the degree of positive immunohistochemical stainings.

**Table 2:** Epidrugs in TGCT and other cancers.

Pharmacologic Group	Drugs	TGCT		Ref	Other Malignancies
		Studies			Indications
		Cell lines	Patients		
DNMTi	5-aza	X	X		Myelodysplastic syndromes
	5-aza-cD	X	X		
	Guadecitabine		X		
KDMi	Chaertocin			[141]	
	JIB-04			[141]	
EZH2i	Tazemetostat				Follicular NH lymphoma
Bromodomaini	JQ11	X		[143]	
LSD1i	CBB3001	X			
HDACi	belinostat	X		[144]	Cutaneous T cell lymphoma
	chicamide	X			
	depsipeptide	X		[144]	
	panobinostat	X		[144]	Cutaneous T cell lymphoma
	Romidepsin	X			Cutaneous T cell lymphoma
	Quisinostat	X		[141]	
	trichostatin				
	vorinostat	X			
CDKi	dinaciclib			[140]	
	Flavo-piridol			[140]	
	NVP-2			[140]	
	SY0351			[140]	
	thal-sns-032			[140]	
	THZ1			[140]	
	THZ531			[140]	
	YKL-5-214			[140]	

## Supplementary Materials

The following supporting information can be downloaded at: https://www.mdpi.com/article/10.3390/ijms24044148/s1.

## Abbreviations

AFP	alfa fetoprotein
5-aza	5-azacytidine
5-aza-cD	5-aza-2′-deoxycytidine
BEP	bleomycin, etoposide, and (cis)platin
bromodomaini	bromodomain inhibitor
CC	choriocarcinoma
CDK	cyclin D kinase
CDKi	CDK inhibitor
CYT	spermatocyte
DNMT	DNA methyltransferase
DATECA	the Danish testis cancer study group
DNMT	DNA methyl transferase
DNMTi	DNA methyltransferase inhibitor
EC	embryonal carcinoma
EGFR	epidermal growth factor receptor
ESC	embryonic stem cells
*EZH2*	*e*nhancer of Zeste-2
EZH2i	EZH2 inhibitor
GCNIS	germ cell neoplasia in situ
GON	gonocytes
HAT	histone acetylase
hCG	human chorionic gonadotropin
HDAC	histone deacetylase
HDACi	histone deacetylase inhibitor
HDMT	histone demethylase
HMT	histone methyltransferase
iPSC	induced pluri- to totipotent stem cells
KDM	lysine demethylase
KDMi	lysine demethylase inhibitor
Kme	methylated histone 3 lysine
LDH	Lactate dehydrogenase
LSD1i	LSD1 inhibitor
5mC	5-methyl cytosine
5mhC	5-methylhydroxy cytosine
MGCT	microinvasive germ cell tumor
NGC	normal germ cells
NSCLC	non-small cell lung cancer
NST	non-seminomatous germ cell tumor
OCT4	the protein of *POU5F1*
OSKM	the *OCT4, SOX2, KLF4,* and *MYC* panel
OSLN	the *OCT4, SOX2, LIN28,* and *NANOG* panel
PGC	primordial germ cells
PRC2	polycomb repressor complex 2
SCLC	small cell lung cancer
SCEC	small cell esophageal cancer
SE	seminoma
SPA	spermatogonia type A
SPB	spermatogonia type B
TER	teratoma
TET	DNA demethylase
TGCA	The Genomic Cancer Atlas
TKI	tyrosine kinase inhibitor
TNM	international tumor, nodes, and metastases classification
TSS	transcription start site.
WHO	World Health Organization
YST	yolk sac tumor
