# Supplementary material for: Epigenetic Regulation of Driver Genes in Testicular Tumorigenesis
Source: Int J Mol Sci. 2023 Feb 19;24(4):4148. doi: 10.3390/ijms24044148 (PMC9966837; doi:10.3390/ijms24044148)
Supplement: Supplementary file 1 [file ijms-24-04148-s001.zip › ijms-2140089-supplementary.pdf]

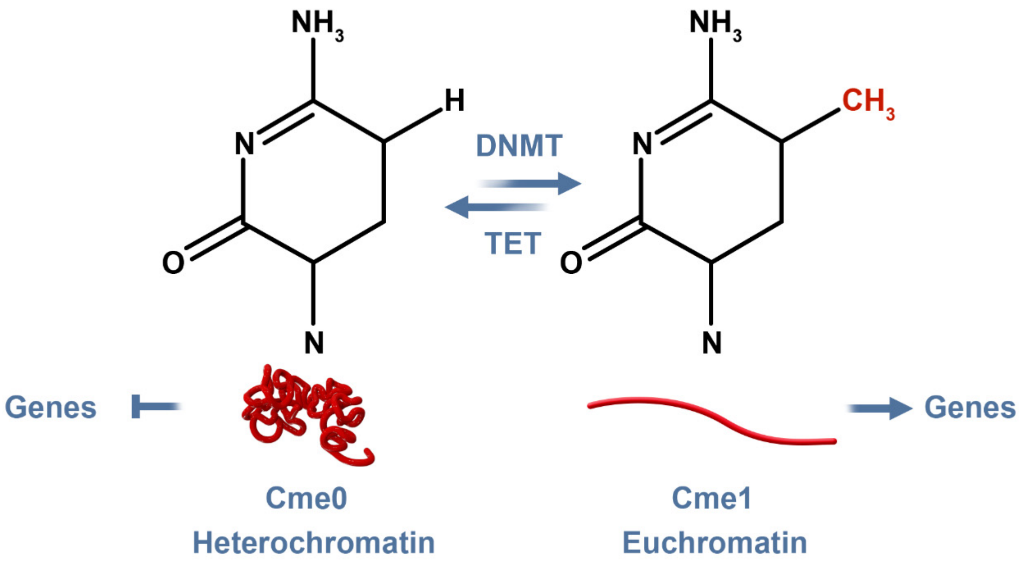

Figure S1. C methylations in the DNA strand in the impact on chromatin structure and gene expression. Abbreviations: DNMT DNA methyltransferase, TET metylcytosinedioxygenase.

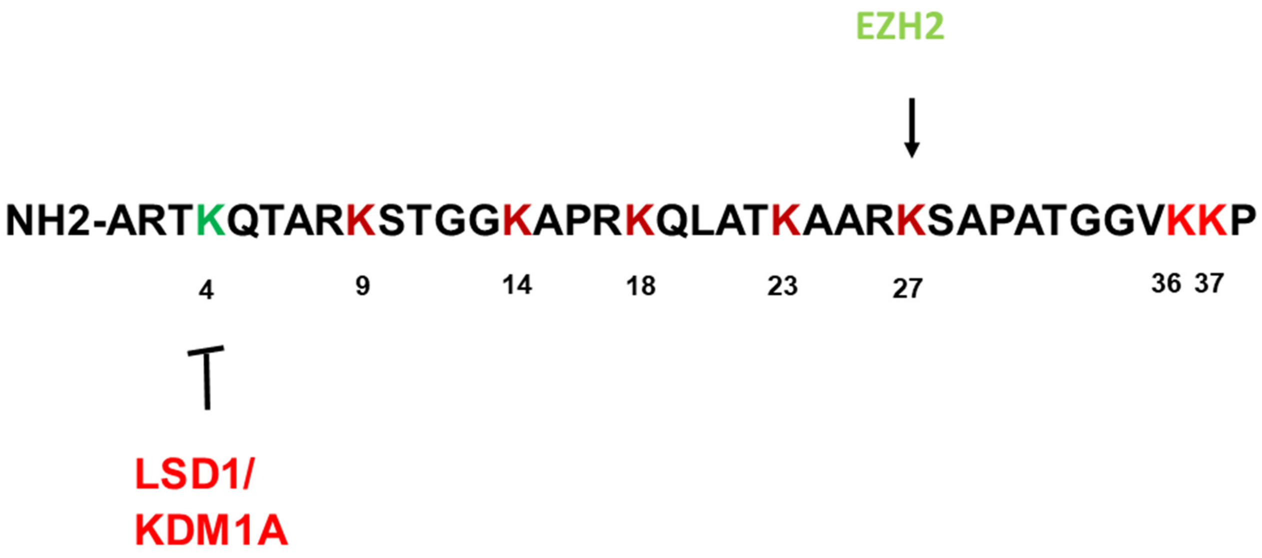

(**a**)

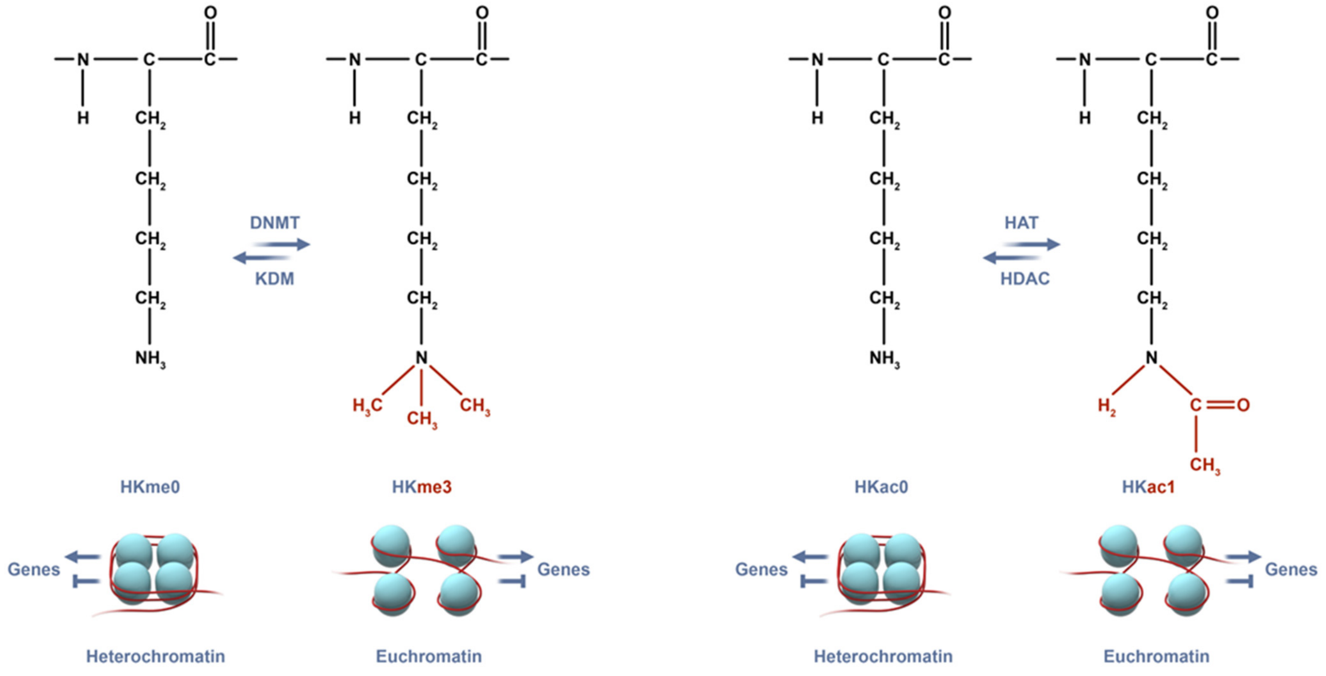

(**b**)

Figure S2. Histone modifications. (**a**) shows the histone 3 amino acid chain. Abbreviations: K is abbreviation for lysine. The figure shows a known metylation and demetylation of histone 3 lysines in TGCT. (**b**). Methylations and acetylations of histone 3 lysines have an impact on chromatin structure and gene expression.
